# Intra-peritoneal rupture of large hydatid cyst of the liver containing innumerable daughter cysts after blunt abdominal trauma

**DOI:** 10.1016/j.ijscr.2019.09.035

**Published:** 2019-10-01

**Authors:** Ayad Ahmad Mohammed, Sardar Hassan Arif

**Affiliations:** Department of Surgery, College of Medicine, University of Duhok, Kurdistan Region, Iraq

**Keywords:** Abdominal trauma, Echinococcus, Hydatid disease, Intraperitoneal rupture, Laparotomy

## Abstract

**Introduction:**

Hydatid disease is a major health concern in the Mediterranean countries and many other parts of the world. The route of transmission is fecal-oral, and then spread by the portal venous system almost all part of the body.

The liver is the most commonly involved organ, followed by the lungs, and other organs are involved in order of decreasing frequency.

**Presentation of case:**

A middle-age male patient presented with acute abdominal pain and repeated vomiting after blunt abdominal trauma. CT-scan showed a large hydatid cyst of the right lobe of the liver with features of intraperitoneal rupture. Emergency laparotomy was performed with extraction of a countless number of daughter cysts from the peritoneal cavity. We further examined the bowel and found no abnormalities. The cyst was not causing any compression on the bile duct. Omentoplasty was performed for the cavity. The patient was discharged home eight days after the operation and the drain removed after twenty-five days when the bile leak stopped, anthelminthic drugs were given for three months after the surgery.

**Conclusion:**

Prevention of hydatid disease is the most effective way to control the disease, the cyst may reach a very large size and be asymptomatic. Intraperitoneal rupture may occur as a result of blunt abdominal trauma which could be trivial.

## Highlights

• Hydatid disease is a real health problem in endemic regions.
• The disease may be discovered incidentally during routine investigations.
• Intraperitoneal rupture is very serious that may result in severe anaphylactic reaction.

## 1 Introduction

Hydatid disease is a major health concern in the Mediterranean countries and many other parts of the world such as New Zealand, Australia, South America, and Asia. It is caused by a tape worm belonging to the *Echinococcus* species, human infection mainly occurs due to 2 types: *Echinococcus granulosus* & *Echinococcus multilocularis* [1,2].

The route of transmission is fecal-oral, the eggs of the parasites are hatched and spread by the portal venous system and lymphatic system to almost all part of the body.

The liver is the most commonly involved organ, followed by the lungs, and other organs are involved in order of decreasing frequency [2,3].

The work of this case report has been reported in line with the SCARE criteria, 2018 [4].

## 2 Patient information

A-46-year old male presented to the emergency department complaining from abdominal pain and repeated bilious vomiting after blunt abdominal trauma due to a car accident.

### 2.1 Clinical findings

**Fig. 1:**
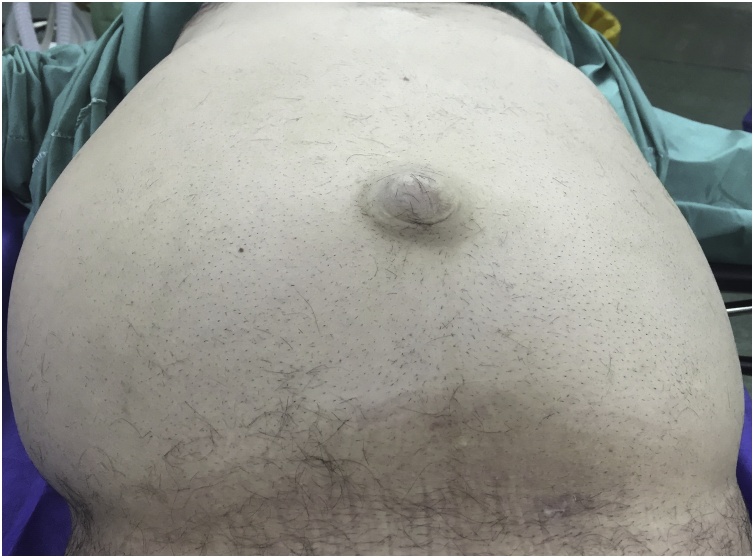
A picture showing the abdomen of the patient with bulging in the right side of the abdomen and umbilical hernia.

The patient was admitted to the emergency ward, during examination the patient had tachycardia with normal blood pressure, the patients had no features of allergic reaction. Abdominal examination showed asymmetrical counter of the abdomen with visible bulging in the right flank, the patient had generalized abdominal tenderness with guarding, the patient had umbilical hernia for three years, Fig. 1. There past medical and surgical histories were negative.

### 2.2 Diagnostic assessment

**Fig. 2:**
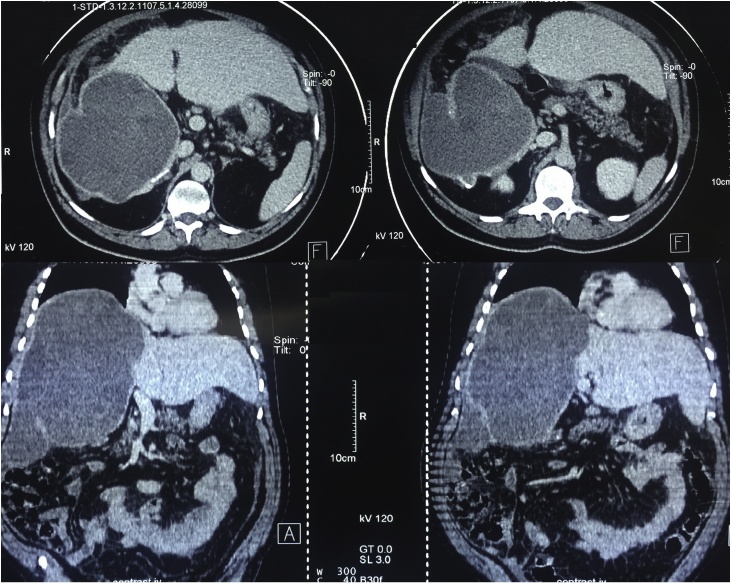
CT-scan of the abdomen showing a large hydatid cyst in the right lobe of the liver with fluid collection around the cyst.

CT-scan of the abdomen showed a large hydatid cyst (24 cm x 14 cm) in the right lobe of the liver, with free fluid in the peritoneal cavity suggesting ruptured of the cyst, Fig. 2.

### 2.3 Therapeutic intervention

**Fig. 3:**
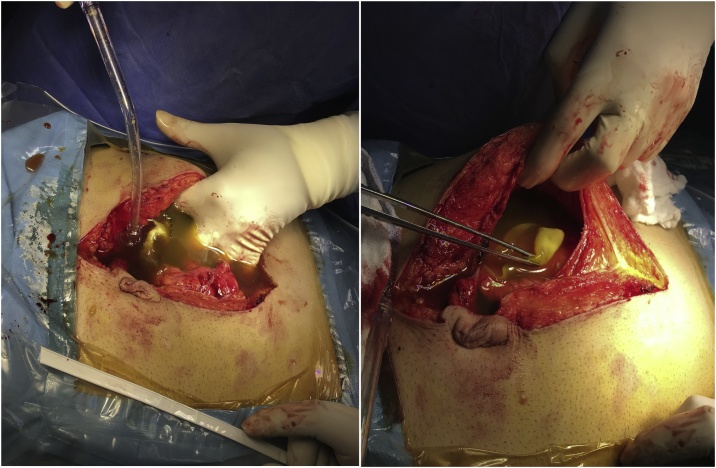
An intraoperative picture showing the peritoneal cavity filled with bile stained fluid with floating daughter cysts.

**Fig. 4:**
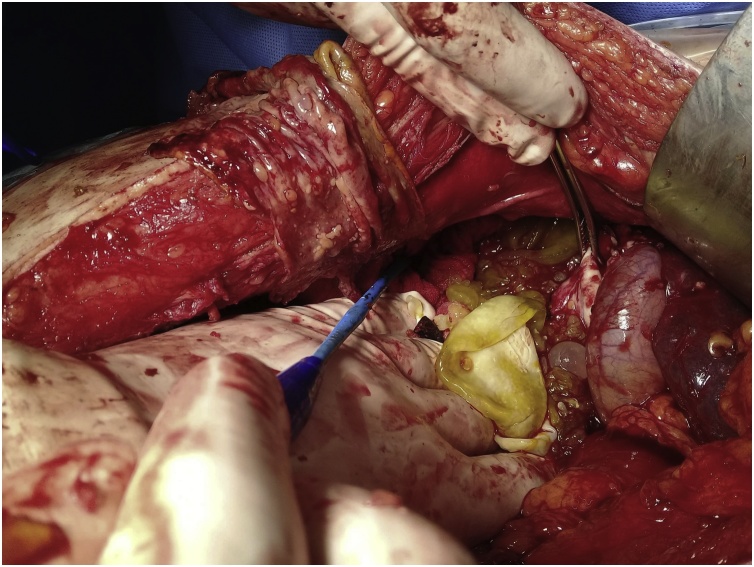
An intraoperative picture showing a large number of daughter cysts in the peritoneal cavity.

During laparotomy the peritoneal cavity was filled with bile stained fluid with multiple floating daughter cysts inside, Fig. 3, Fig. 4.

**Fig. 5:**
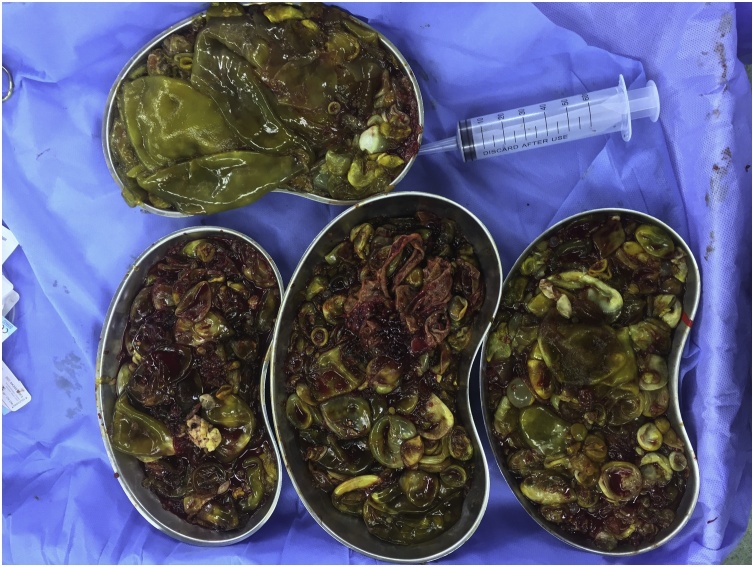
A large number of daughter cysts extracted from the abdomen and the right lobe of the liver.

Aspiration of the fluid was done and extraction of a countless number of daughter cysts from the peritoneal cavity and from the cyst cavity in the right lobe of the liver was done, areas of bile leak from the inside of the cyst were sutured with slowly absorbable suture material, Fig. 5.

### 2.4 Follow-up and outcomes

The patient was discharged eight days later, the drain remained inside for twenty-five days when the bile leak stopped then. The patient received anthelminthic drugs for three months after the operation.

## 3 Discussion

Hydatid cysts of the liver may be discovered incidentally during routine investigations or patients may present with right hypochondrial pain, the presentation of ruptured hydatid cyst include abdominal pain, fever, vomiting, jaundice, and anaphylaxis. Falls are the most common cause of rupture followed by blunt abdominal trauma [5].

Some authors classify cyst rupture into 3 types; contained rupture which occurs when the cyst rupture partially but contained within the host tissue, communicating rupture occurring when the rupture occurs into the biliary tree, and direct rupture when the rupture occurs to the peritoneal cavity. Rupture of the cyst should be treated with urgent surgery especially the direct one combined with adjuvant medical treatment to decrease the risk of peritoneal hydatidosis [6].

Ultrasound is one of the most useful tools in diagnosing this disease in the liver. CT-scan and MRI add more anatomical details about the cyst, the presence of membrane inside, hydatid sand, daughter cysts, calcifications, and communication with the biliary tree, CT-scan in the most useful diagnostic tool in diagnosing intra-peritoneal cyst rupture. Serological tests also may help in the diagnosis [2,5].

Hydatid disease should be one of the differential diagnoses in any cystic lesion of the liver and other organs [7].

The treatment of hydatid cyst of the liver depends on the presentation and the presence of complications that are associated with the cyst [1].

Medical treatment with anthelminthic drugs such as albendazole was effective in some patients regardless to the size of the cyst [8].

Complications of hydatid disease include intra-peritoneal or intra-biliary rupture, rupture into the lumen of the bowel, trans-diaphragmatic chest involvement and involvement of the portal veins. Death may occur in some complicated cases or from anaphylaxis [9].

Intra-biliary rupture of the hydatid cyst may occur and the patient may present with jaundice or cholangitis, the presence of bile stained fluid inside the cyst indicates communication with the biliary tree, trans-duodenal sphincterotomy or T-tube drainage may be done with excision of the liver cyst or partial liver resection [10].

Recurrence of the cyst may occur and may be due to previous rupture of the cyst, failure to remove or kill all the viable cysts and protoscolices. It is very important to use meticulous technique during operation to prevent spillage of the contents to the peritoneal cavity to prevent a recurrence [11,12].

## Patient’s perspective

After the car accident, I was afraid of immediate death because I had severe abdominal pain and it was strange for me that the first times the cyst in the liver was discovered after this accident.

## Sources of funding

None.

## Ethical approval

Ethical approval has been exempted by my institution for reporting this case.

## Consent

Written informed consent was obtained from the patient for publication of this case report and accompanying images.

## Author’s contribution

Dr Ayad Ahmad Mohammed and Dr Sardar Hassan Arif are the surgeons who performed the operation.

The concept of reporting the case, data recording, and drafting the work done by Dr Ayad Ahmad Mohammed and Dr Sardar Hassan Arif

Dr Sardar Hassan Arif took the consent from the patient for publishing the case.

Final approval of the work to be published was done by Dr Ayad Ahmad Mohammed.

## Registration of research studies

This work is case report and there is no need of registration.

## Guarantor

Dr Ayad Ahmad Mohammed is guarantor for the work.

## Provenance and peer review

Not commissioned, externally peer-reviewed.

## Declaration of Competing Interest

The author has no conflicts of interest to declare.

## References

[1] Dziri C. (2001). Hydatid disease-continuing serious public health problem: introduction. World J. Surg..

[2] Mohammed A.A., Arif S.H. (2019). Hydatid cyst of the parietal peritoneum. J. Pediatr. Surg. Case Rep..

[3] Mohammed A.A., Arif S.H. (2019). Hydatid cyst of the calf presenting as painless mass; a case report. Int. J. Surg. Case Rep..

[4] Agha R.A. (2018). The SCARE 2018 statement: updating consensus Surgical CAse REport (SCARE) guidelines. Int. J. Surg..

[5] Gunay K. (1999). Traumatic rupture of hydatid cysts: a 12-year experience from an endemic region. J. Trauma Acute Care Surg..

[6] Lewall D.B., McCorkell S.J. (1986). Rupture of echinococcal cysts: diagnosis, classification, and clinical implications. Am. J. Roentgenol..

[7] Arif S.H., Mohammed A.A. (2018). Primary hydatid cyst of the urinary bladder. BMJ Case Rep..

[8] Saimot A. (1983). Albendazole as a potential treatment for human hydatidosis. Lancet.

[9] Papadimitriou J., Mandrekas A. (1970). The surgical treatment of hydatid disease of the liver. Br. J. Surg..

[10] Paksoy M. (1998). Rupture of the hydatid disease of the liver into the biliary tracts. Dig. Surg..

[11] Sielaff T.D., Taylor B., Langer B. (2001). Recurrence of hydatid disease. World J. Surg..

[12] Mohammed A.A., Arif S.H. (2019). Surgical excision of a giant pedunculated hydatid cyst of the liver. J. Surg. Case Rep..

